# Adaptive evolution of proteins in hepatitis B virus during divergence of genotypes

**DOI:** 10.1038/s41598-017-02012-8

**Published:** 2017-05-16

**Authors:** Shengdi Li, Zhen Wang, Yixue Li, Guohui Ding

**Affiliations:** 1 Key Laboratory of Computational Biology, CAS-MPG Partner Institute for Computational Biology, Shanghai Institutes for Biological Sciences, Chinese Academy of Sciences, 320 Yue Yang Road, 200031 Shanghai, China; 2 University of Chinese Academy of Sciences, 19 Yuquan Road, Beijing, 100049 China; 3 Shanghai Center for Bioinformation Technology, Shanghai Industrial Technology Institute, 1278 Keyuan Road, 201203 Shanghai, China

## Abstract

Hepatitis B virus (HBV) is classified into several genotypes, correlated with different geographic distributions, clinical outcomes and susceptible human populations. It is crucial to investigate the evolutionary significance behind the diversification of HBV genotypes, because it improves our understanding of their pathological differences and pathogen-host interactions. Here, we performed comprehensive analysis of HBV genome sequences collected from public database. With a stringent criteria, we generated a dataset of 2992 HBV genomes from eight major genotypes. In particular, we applied a specified classification of non-synonymous and synonymous variants in overlapping regions, to distinguish joint and independent gene evolutions. We confirmed the presence of selective constraints over non-synonymous variants in consideration of overlapping regions. We then performed the McDonald-Kreitman test and revealed adaptive evolutions of non-synonymous variants during genotypic differentiation. Remarkably, we identified strong positive selection that drove the differentiation of *PreS1* domain, which is an essential regulator involved in viral transmission. Our study presents novel evidences for the adaptive evolution of HBV genotypes, which suggests that these viruses evolve directionally for maintenance or improvement of successful infections.

## Introduction

The hepatitis B virus (HBV) is one of the most prevalent viral infections worldwidely^1, 2^ and is known as a leading cause of liver diseases. The viral genome is a circular, partially double-stranded DNA of ~3.2 kb and comprises 4 open reading frames (ORFs) encoding 7 proteins^3, 4^: 1) *P* ORF encodes the polymerase; 2) *PreC*/*C* ORF encodes capsid proteins; 3) *PreS1*/*PreS2*/*S* ORF encodes large (L), middle (M) and small (S) surface proteins; and 4) *X* gene encodes the secretary X protein. Overlapping genes comprise ~50% of the entire HBV genome. This feature has been suggested to constrain their evolution, as evidenced by decreased nucleotide diversity in overlapping regions vs. non-overlapping regions^5, 6^. In HBV, *PreS1/PreS2/S* ORF is completely overlapped by *P* ORF.

To date, 10 HBV genotypes (A–J) have been identified based on the criteria of >8% genetic differences in the genome sequence and >4% in *S* gene^7, 8^. Epidemiology of HBV infections has revealed distinct geographic and ethnic distributions of HBV genotypes^9, 10^. Among the 8 major genotypes (A–H), genotypes A and D primarily spread in Europe and Africa, genotypes B and C are commonly found in Asia, genotype E is prevalent in central and western Africa, genotypes F and H are restricted to Latin America and Alaska, and genotype G is reported in Europe and the United States^10, 11^. The genetic differences among HBV genotypes also affect clinical outcomes, drug responses and main transmission routes^10, 11^. The origin of HBV genotypes remains highly controversial. There are conflicting hypotheses of the exact origin time^12–16^, while the main contradiction lies in the disagreement of evolutionary rates^11^. A recent opinion on this debate explained an inconstant evolutionary rate of HBV genotypes between short-term and long-term events, depending on their main transmission routes and dynamics of the infected populations^11^.

Many efforts have been made to understand the evolution of HBV by measuring selective pressures acting on the viral proteins^17–20^. A recent approach suggested positive selection signals of HBV were associated with disease stages and viral genotypes^17^. In particular, the joint evolution of *PreS1/PreS2/S* ORF and *P* ORF has attracted significant attentions^18–20^. A strict selection is reported for the *PreS1* domain^19^, while its overlapping region, so called the *Spacer* domain of *P* ORF, is relaxed and prone to non-synonymous mutations^19, 20^.

In general, our current understanding of the selective pressures over the HBV proteins is mainly based on the ratio of non-synonymous vs. synonymous substitution rate *d*_N_/*d*_S_^21^, which is a commonly accepted statistical method of testing neutrality for viral protein variants^6, 17–20, 22^. However, we argue that using the *d*_N_/*d*_S_ statistic for HBV has a few limitations. First off, the power of *d*_N_/*d*_S_ ratio to indicate the direction of selection in overlap genes is under question. The underlying assumption using *d*_N_/*d*_S_ ratio to imply positive or negative selection is to treat *d*_S_ as the neutrally evolving rate^21^, which is in conflict with the fact that synonymous sites of overlapping genes often cause non-synonymous alterations of another ORF. Further support to this statement is the decreased synonymous substitution rate observed in overlapping vs. non-overlapping regions of HBV^5^. Second, the outcome of the codon-based statistic regarding overlapping genes reflects a mixture of independent and joint evolutions of multiple ORFs, therefore it cannot distinguish the exact selection pressure over single gene. A solution to this problem is to take into account independently evolving sites, as the evolutions of overlapping genes are largely independent at specific codon positions^18^. Third, *d*_N_/*d*_S_ represents an average substitution rate ratio over the entire phylogeny of input strains, regardless of defined genetic groups such as genotypes. However, the evolution of HBV is regarded to be dynamic between short-term and long-term events^11^. Thus, alternative methods considering the inconsistency of genotypic evolutions will provide novel information to the standing questions.

In the present study, we applied stringent criteria to collect HBV genome sequences representing 8 major genotypes (A–H) from public database. We used a specified definition of protein-altering and neutral variants in consideration of gene overlaps to investigate inner-genotypic polymorphisms and inter-genotypic fixations. We applied the McDonald-Kreitman (MK) test and its related statistics^23, 24^ to our HBV dataset to examine whether positive selection drives the differentiation of protein-coding genes between genotypes. Through the present study, we aimed to provide a novel insight into HBV protein evolution in presence of natural selection.

## Results

### Construction of HBV genomic dataset of eight genotypes

To study the genotypic diversity of HBV, we constructed a dataset of 6765 HBV genome sequences, which is approximately the entire public collection after exclusion of redundancy and poor quality (See Materials and Methods). We subsequently used a fragment typing approach^25, 26^ to predict genotypes of the sequences and to remove putative inter-genotypic recombinants (n = 2713) (Supplementary Table S1), thereby ensuring the remaining sequences consist of no admixture between genotypes. To validate the method, we randomly selected three sequences from each genotype (in total 24 pure strains), as well as 10 sequences characterized as putative recombinants (Supplementary Table S2). Then, we analyzed them using jpHMM, which is a probabilistic model-based method for predicting inter-genotypic recombinants^27^. All 24 pure strains, as well as 8 inter-genotypic recombinants were characterized by both genotyping methods (Supplementary Table S2, Supplementary Figure S1). Although 2 out of the 10 recombinants were identified with ambiguous recombination events (FJ361772, KJ586811), the consistent predictions of randomly selected pure strains by both methods suggested good quality of the remaining dataset (Supplementary Table S2). After exclusion of inter-genotypic recombinants, we filtered the remaining dataset to remove strains infecting non-human primates (n = 105) (Supplementary Table S3), strains with insertion/deletion polymorphisms (n = 941) and population outliers (n = 14) (Supplementary Table S4, Supplementary Figure S2). 2992 sequences from eight genotypes (A-H) remained after all steps of sample exclusion (Supplementary Table S4).

**Figure 1:**
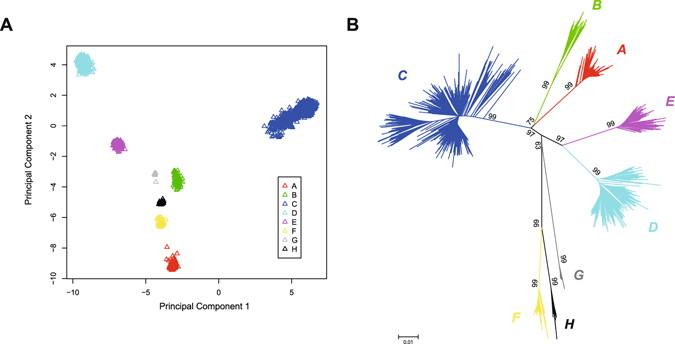
Population structure of 2992 HBV genomes. (**A**) The Principal Component Analysis of 2992 HBV genome sequences, which were labeled in different colors according to their genotypes predicted by fragment typing. (**B**) The unrooted phylogeny tree was computed using Neighbor-Joining method. 100-time bootstrapping test was performed and showed as numbers on clade.

The remaining samples showed a clear division of genetic background with respect to their genotypes. In the Principal Component Analysis (PCA)^28^ of all 2992 genomes, strains from each genotype were clustered and no obvious admixture between clusters was observed (Fig. 1A). Phylogenetic analysis also confirmed the classification: the root branches of each clustered genotype sub-clade were robust under bootstrapping test and no outlier was observed (Fig. 1B). Given these, we applied the 2992 sequences to our further analysis as the final dataset, which represented distinct and unmixed genetic groups.

### Selective constraints on genome of HBV from different genotypes

**Figure 2:**
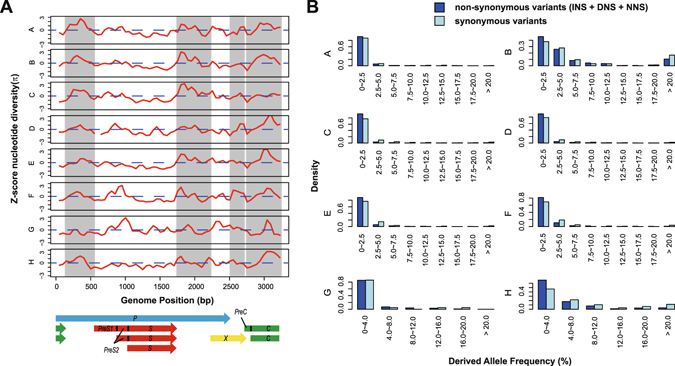
Polymorphisms within HBV genotypes in presence of constraints. (**A**) Z-transformed pair-wise nucleotide difference Z_*π*_ was displayed for eight genotypes (A–H) over a 100-bp sliding window with a step size of 50 bp. Regions without gene overlaps are highlighted with grey background. (**B**) Distribution of derived allele frequency (DAF) of non-synonymous variants and synonymous variants, where non-synonymous variants comprise INS, DNS and NNS variants. In particular, histogram of genotypes A–F were showed in a high resolution with more separate intervals of variant frequencies (each bar represents an interval of 2.5%), while distributions in genotypes G, H were showed in low resolution (4%) because of small sample sizes.

With the 2992 genome dataset, we next examined the nucleotide polymorphisms within eight genotypes to see whether the inner-genotypic evolution of HBV was constrained by selection. The extent of polymorphisms on different genomic regions was measured by the Z-transformed pair-wise nucleotide difference Z_*π*_, using a 100-bp sliding window with a step size of 50 bp (See Materials and Methods). The highly diverse regions, referring to the peaks on curves of Z_*π*_, were mostly correlated with non-overlapping regions (Fig. 2A), indicating presence of constraints on regions with dual ORFs. A general reduction of diversity on overlapped regions, including the *S* vs. *P* and *X* vs. *P*, were observed in genotypes A–F and H. Specifically, there was a peak at *X* vs. *P* region for genotype G, but we found its sample number (n = 27) and genetic variation too small for giving a robust estimation. Moreover, an exceptional peak was observed at the overlapping region *PreS* vs. *P*, correlated with the fact that the *Spacer* domain of *P* ORF within this region is mutation-prone^19, 20^. Interestingly, this signal exhibited a genotype-specific pattern, which is dominant in genotype F and G, and slight or absent in other genotypes (Fig. 2A).

As we described in the former section, our current understanding of selective pressures acting on HBV is controversial in respect of overlapping gene evolution. To solve this problem, we proposed a specified definition of variant types regarding overlapping regions. Briefly, we defined three types of non-synonymous variants and one type of synonymous variants: 1) non-overlapping non-synonymous (NNS) variants; 2) independent non-synonymous (INS) variants, which is present in overlapping regions and causes only one amino acid alteration; 3) dual non-synonymous (DNS) variants, which causes amino acid mutations of two overlapping genes; and 4) synonymous variants, which consist of both overlapping and non-overlapping mutations that cause no protein alterations (An example of determining 4 types of variants was showed in Supplementary Figures S3 and S4). In particular, we have referred to the previously described concept of independently evolving sites^18^ when we defined INS variants, nevertheless the two definitions are still different as INS variants are not restricted to a specific codon position. More concretely, the independently evolving site of *S* ORF is the *P*3/*S*2 codon position^18^, whilst the *P*3/*S*2 site contains both INS and DNS variants of *S* gene. In most cases, INS variant is a better estimator for independent gene evolution, as a minority of variants at independently evolving sites (like *P3/S2*) are jointly non-synonymous mutations of multiple ORFs.

Based on the new classification of variants, we next compared the distribution of allele frequencies between non-synonymous (INS + DNS + NNS) and synonymous mutations. The frequency of a variant (allele) was defined as the proportion of strains carrying the specific mutation within a genotype (See Materials and Methods). According to the histograms, more non-synonymous variants than synonymous variants were found to be singletons or enriched at low frequencies (Fig. 2B). This feature indicates that the non-synonymous variants of HBV genome tend to be preserved at low genetic diversities compared with synonymous variants, suggesting an overall strict selection (on average) acting on the HBV proteins within genotypes.

### Evolution of non-synonymous variants in genotypic differentiation

To figure out whether the diversification of HBV genotypes is driven by natural selection, we subsequently performed the MK test and computed its related statistic *α* and *AS* (see Materials and Methods). Briefly, the MK test measures positive selection by comparing the fixed/polymorphic mutation count ratio between putatively selected sites (e.g. non-synonymous sites) and synonymous sites. In the case of the present study, the fixed mutations were defined as inter-genotypic differences, and the polymorphic mutations were defined as inner-genotypic variations (The method was described in Materials and Methods, and examples of determining fixed and polymorphic mutations were showed in Supplementary Figures S3 and S4).

**Figure 3:**
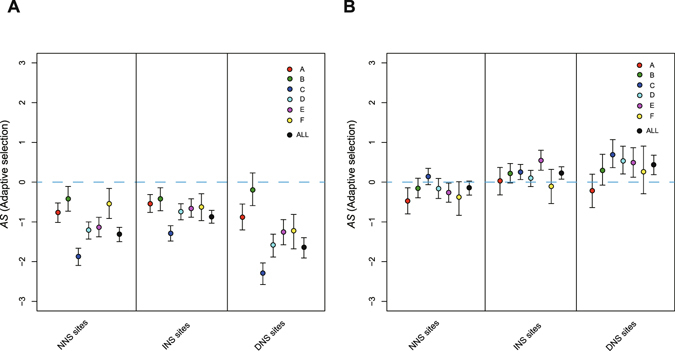
Adaptive evolution of non-synonymous variants in HBV. (**A**–**B**) Plots of *AS* statistics of NNS, INS and DNS variants. The calculation was performed using (**A**) low frequency and (**B**) high frequency variants separately. Low frequency variants were defined by <1% DAF or singletons. *AS* was first computed based on polymorphic variants in each genotype separately and then all polymorphic variants were combined (dot in black). Genotypes G and H are not showed because of small sample size (Supplementary Tables S4 and S5). Error bars denotes the 90% confidence intervals derived from bootstrapping tests of 1000 times for *AS*. Blue dashed line denotes the neutral index, where *AS* = 0.

One common concern of the MK test is that the presence of slightly deleterious mutations will downwardly bias the estimation of positive selection. This effect can be reduced by excluding variants at low frequencies, although the underestimation still exist^29^. However, in our data, the power of the MK test to detect positive selection is satisfactory. As expected, the level of adaptive selection *AS* in low-frequency non-synonymous variants (Derived Allele Frequency < 1% and singletons) is much lower than neutral sites (*AS* < 0) (Fig. 3A). After excluding these variants, the number of fixed mutations is lower than or nearly neutral in NNS sites (*AS* = −0.145), but slightly higher in INS (*AS* = 0.236) and DNS (*AS* = 0.438) sites (Fig. 3B, Table 1). Meanwhile, the estimation of *AS* is more consistent among genotypes in high-frequency variants, indicating a relatively constant ratio of non-synonymous to synonymous substitution rate among different genotypes (Fig. 3B, Supplementary Table S5). By *χ* ^2^ tests^30^ over the 2 × 2 mutation count table regarding three types of non-synonymous sites (See Materials and Methods), we identified the signature of *AS* which showed statistical significance in DNS variants (P-value = 0.0114), close to significance in INS variants (P-value = 0.0651), and no significance in NNS variants (P-value = 0.2735) (Table 1). It should be noted that the heterogeneity of *AS* among DNS, INS and NNS variants did not prove that adaptive evolution is determined by the type of variants, because the three types of non-synonymous variants distributed differently among proteins. The essential conclusion is that adaptive evolution does affect the fixation of non-synonymous variants, and is likely enriched in overlapping regions because DNS and INS show more positive *AS* value than NNS variants. Moreover, the extent of adaptive evolution can be even higher than expected from *AS* value, because this estimator is downwardly biased in presence of slightly deleterious mutations.

**Table 1:** MK test and *AS*, *α* statistic of different categories of variants.

Variant type	Fixed mutation	Polymorphic mutation	*AS*	MK test’s *α*	P value (*χ* ^2^ test)
synonymous	383	1450	/	/	/
NNS	242	1013	−0.145	−0.106	0.2735
INS	288	926	0.236	0.151	0.0651
DNS	121	338	0.438	0.262	0.0114

### Diversification of HBV genes driven by positive selection

With respect to their functions, proteins of HBV contribute differently to viral fitness, therefore the proportions of their non-synonymous variants under positive selection are heterogeneous. To figure this out, we performed the MK test and computed statistic *AS* for each gene separately. Note that the independent adaptation of a gene was estimated based on its NNS and INS variants (or INS variants if the gene is completely overlapped), because evolution of DNS variants are affected by selection over multiple genes.

**Figure 4:**
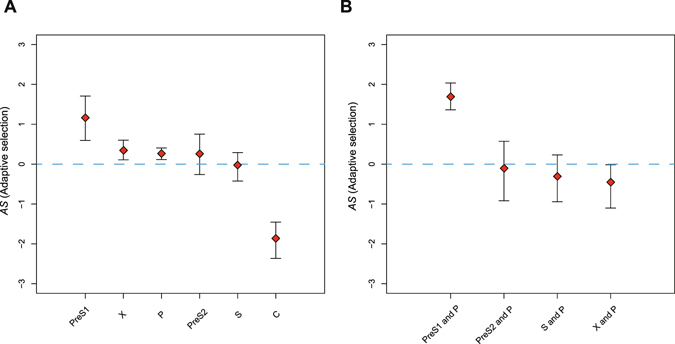
Adaptive evolution of protein-coding genes in HBV. Plots of *AS* statistics for protein-coding genes in HBV. (**A**) Independent gene evolutions were estimated based on NNS + INS or INS (*PreS1/PreS2/S* contains no NNS variants) variants, and (**B**) co-evolutions of overlapped genes were estimated with DNS variants, using all observed mutations from eight genotypes. Genes or regions with few observations of fixed or polymorphic mutations (<10 mutations) are not showed (Table 2). Error bars indicate 90% confidence intervals and blue dashed line denotes neutral index similar as in Fig. 3.

Interestingly, we detected a predominant signal of adaptive evolution in *PreS1* which encodes the N-terminal extension only in L surface protein (Fig. 4A). >50% of its differentiated INS variants among genotypes (*AS* = 1.168, *α* = 0.555, P-value = 0.0049) are driven by positive selection (Table 2). Meanwhile, the *PreS2* and *S* genes didn’t show significantly elevated number of non-synonymous fixations (*PreS2*: *AS* = 0.265, *α* = 0.168, P-value = 0.4839; *S*: *AS* = −0.024, *α* = −0.017, P-value = 0.9283), suggesting that the significant signature is restricted to L protein, but not M and S proteins. Even on their DNS sites, where the L, M or S surface proteins jointly evolve with the polymerase, similar signature was found only in *PreS1* (*AS* = 1.685, *α* = 0.689, P-value = 2.73^−12^) (Fig. 4B, Table 2). This is explained by the fact that *PreS1* is largely overlapped with the less functionally relevant *Spacer* domain of *P* ORF^31^, thus evolution of DNS sites on *PreS1* is less affected by gene overlaps.

**Table 2:** MK test and *AS*, *α* statistic of variants in genes.

Variant type	Gene	Fixed mutation	Polymorphic mutation	*AS*	MK test’s *α*	P value (*χ* ^2^ test)
synonymous	/	383	1450	/	/	/
INS	*PreS1*	19	32	1.168	0.555	0.0049
INS	*PreS2*	20	63	0.265	0.168	0.4839
INS	*S*	40	154	−0.024	−0.017	0.9283
NNS + INS	*P*	322	1013	0.267	0.169	0.0312
NNS + INS	*X*	100	299	0.340	0.21	0.0669
NNS + INS	*PreC*	3	22	−0.954	−0.937	0.2762
NNS + INS	*C*	26	356	−1.855	−2.617	1.08E-10
DNS	*PreS1* and *P*	68	80	1.685	0.689	2.73E-12
DNS	*PreS2* and *P*	13	53	−0.107	−0.077	0.814
DNS	*S* and *P*	20	94	−0.312	−0.241	0.3915
DNS	*X* and *P*	12	62	−0.449	−0.365	0.3302
DNS	*PreC* and *X*	2	9	−0.247	−0.187	0.8253
DNS	*C* and *P*	6	40	−0.816	−0.761	0.1943

The *PreS1/PreS2/S* ORF is completely overlapped by *P* ORF, therefore *PreS1, PreS2, S* contains no NNS variants.

Given the remarkable signature of adaptive evolution on *PreS1*, we next analyzed its 119 amino acid positions to search for fixed amino acid differences among genotypes. In particular, we identified 47 positions where at least one genotype possessed an alternatively dominant amino acid, and characterized a total of 76 alternative amino acid variants (Supplementary Table S7). This number is slightly lower than that of nucleotide variants (INS + DNS in *PreS1*: 19 + 68 = 87 nucleotide variants), because some amino acid changes comprise more than one step of non-synonymous nucleotide substitutions. We showed in Table 3 the genotype-specific amino acids of *PreS1*, which were mutated and became dominant in 3 or less out of the 8 genotypes. According to the definition of the MK’s *α* statistic, *α* of *PreS1* INS variants equals 0.555 and DNS variants equals 0.689, suggesting that ~55–70% of the differentiated amino acids (42–53 out of the 76 variants), were driven by positive selection. Thus, the differentiated amino acids of *PreS1*, which we listed here, comprise a number of potential genetic determinants of HBV fitness.

**Table 3:** Genotype-specific amino acid variants in *PreS1* region.

Genotype^a^	Typical amino acid variants on *PreS1* ^b^
A	48I, 54A, 67L, 74I, 89S, 90T, 91I
B	35K, 39E, 45L, 48H, 56H, 87S
C	10Q
D	39A, 51T, 65L, 86Q, 91N, 114N
E	16H, 19T, 39R, 45H, 53T, 84M, 86K, 109T
F	8T, 47K, 84V
G	25L, 48K, 51P, 81S, 84T
H	8A, 47T, 88S, 90P
A + B	10K
A + C	35G, 57Q
B + D	54D, 108L
C + D	60A
D + G	19S
E + G	3L, 4S, 5W, 6T, 7V, 9L, 10E, 11W, 14K, 63Y
F + H	3A, 4P, 5L, 7T, 10R, 33L, 39S, 40S, 54M, 62G, 100R, 104K, 108V
A + B + C	3G, 4W, 5S, 7K, 14T, 88V
A + C + E	51H
A + C + G	39N, 115S
B + F + H	51N
C + E + G	54E
D + E + G	18T, 38T
D + F + H	14Q

^a^The viral genotype(s) and their specific *PreS1* amino acid residues are listed. Only genotype-specific variants in three or less genotypes are showed.

^b^The genotype-specific amino acid variants are represented by their positions on *PreS1* peptide (from 1st to 119th residues) and the amino acid symbol.

The other signatures of positive *AS* were found in *P* and *X*. Whilst the absolute *AS* values are approximate in *PreS2* (*AS* = 0.265), *P* (*AS* = 0.267) and *X* (*AS* = 0.340), we found that they were of statistical significance in *P* (P-value = 0.0312), close to significance in *X* (P-value = 0.0669) but no significance in *PreS2* (P-value = 0.4839). Given these results, we assume that the evolutions of *P*, *X* and *PreS2* are driven by slightly positive selection, however only the longest *P* gene has accumulated adequate mutations to reach the power of a statistical test.

In contrast, *C* gene represents the most conserved part of the genome during genotypic differentiation, with the most negative *AS* value (*AS* = −1.855, P-value = 1.08^−10^) among all tested sites (Fig. 4A, Table 2). It is controversial whether *C* gene indeed has less number of fixed variants than neutral sites, because *AS* is possibly underestimated. What is certain, however, is that *C* gene shows much less tendency to undergo adaptive evolution than *PreS1/PreS2/S*, *P* and *X* genes. The most negative *AS* value suggests that *C* gene is the least related to viral adaptations among all HBV genes, therefore the sequence tends to remain stable among lineages.

Based on these findings, we concluded that the *PreS1* domain has experienced adaptive evolution during differentiation of HBV genotypes, evidenced by elevated numbers of fixed INS and DNS variants, while the *C* gene remains conserved. Although the overlap of *PreS1* and the *Spacer* domain gives rise to more flexibility in their DNS variants, it cannot fully explain the increasing proportion of fixed INS variants for *PreS1*. Our results suggest that the function of L surface protein is crucial for the evolution of HBV genotypes.

## Discussion

The initial step of HBV entry is attachment to the membrane of hepatocytic cell. This process is mediated by the interaction between the *PreS1* domain of L protein and the HBV entry receptor of human hepatocytes, namely *NTCP* ^32^. In particular, the N terminal 75 or 77 amino acids of *PreS1* are responsible for its activity in viral infection^33, 34^. On a different note, the *PreS* domain (*PreS1* + *PreS2*) also plays an essential role in the interaction with host immune response, as it contains several immunogenic T- and B- cell epitopes^35–38^. Deletion mutations in the *PreS1* domain are correlated with occult HBV infections (surface antigen level in serum is undetectable) in genotype C^39, 41^, which is regarded as a potential mechanism to escape clearance mediated by immune cells^41^. Given these critical functions in viral infections, our data shows that *PreS1* is of vital importance in genotypic evolutions, represented by a high *AS* signature. Indeed, both attachment to hepatocytes and survival under immune response are highly correlated with viral fitness, to ensure successful infection in human hosts.

Besides the adaptive feature of *PreS1* domain, another interesting finding of out study is the conservation of *C* gene among genotypes. The *C* gene encodes nucleocapsid protein, which serves as the container of partially double-strand DNA and is enveloped by three types of viral surface proteins (L, M, S proteins)^42^. After entry into the cytoplasm, the capsid is released from the envelope and transported towards the nucleus^43^. Its interaction with host is relatively weak, as the mature capsid (DNA-containing) is merely exposed after successful invasion. Thus, *C* gene exhibits a trend to preserve its sequence between heterogeneous viral genotypes, probably because it is not directly involved in adaptation to host.

The advantageous strains of a viral species are ones that survive better in the host and show higher efficiency of replications and infections. The signature of adaptive evolution in *PreS1* suggests that HBV gains fitness potentially through improving infections or evading immune responses. Nevertheless, the remaining questions are what exact adaptation HBVs have experienced and what outcomes have been resulted from different *PreS1* sequences. One explanation for the diversification of *PreS1* domain is that the humans correlated with each viral genotype adapt to infections differently (e.g. by acquirement and fixation of different resistant mutations), thus the viruses evolve accordingly and constantly into diverse directions. This hypothetic model is an application of the classical evolutionary theory called “Red Queen hypothesis”^44^, which proposes an ever-existing evolutionary race between two counter-organisms caused by the conflict of fitness. This theory is also successful in describing the process where virus diverges to infect different mammal species^45^. Regarding the functions of *PreS1*, its strong signature of selection suggests an ever-changing adaptation of the viruses merely to ensure successful infection, as the defensive mechanisms of hosts might also be updated constantly. However, a limitation of this hypothesis is the lack of proof that humans have carried HBV-resistant mutations as a consequence of adaptive process, parallel with HBV genotypic divergence. An association study of Chinese population has surveyed common variants in regulatory regions of *NTCP*, but failed in finding correlation with HBV susceptibility^46^. Several genome wide approaches have discovered in Asian populations that variants of human leukocyte antigen (HLA) DP loci affect HBV infections^47–51^, but it remains unclear whether alleles correlated with better clinical outcomes are favored by positive selection.

Another possible outcome of *PreS1* differentiation is correlated with the change of viral transmission route. The shift of main transmission route (vertical vs. horizontal), which HBV genotypes have experienced, is regarded as a consequence of geographic and demographic features of their infected populations^10, 11^. More concretely, genotypes in endemic regions (e.g. genotype B, C) mainly spread through perinatal or vertical transmission, whilst genotypes widely spreading in and between continents (e.g. genotype A, D) often transmit horizontally^10, 11^. The findings of our study now raises a thoughtful question: is this shift an adaptive process? As commonly expected, traits that favor vertical transmission show advantages in restricted human populations, while qualities that assist horizontal infections are beneficial in epidemic regions. Meanwhile, a signature of positive selection should be present on their genetic determinants. Our data shows that *PreS1* exhibited the highest extent of adaptive evolution compared with other HBV genes. Although *PreS1* is directly correlated with viral infection, it remains unclear whether the L protein or other HBV proteins contribute to the preference of transmission route among genotypes. A future direction regarding this issue is to look for the genetic determinants of vertical- and horizontal-transmission preference in HBV if they do exist.

An additional contribution of this study is the application of a new definition of protein-altering and neutral variants of overlapping genes, by which the independent or joint gene evolutions could be clearly described using statistical test of neutrality. Based on Nei and Gojobori’s *d*_N_/*d*_S_ statistic^21^, a number of studies have reported a general trend for evolution of overlapping regions in virus: one reading frame is subject to strict selection (*d*_N_/*d*_S_ < 1), whilst the other reading frame underlies relaxed selection (*d*_N_/*d*_S_ > 1)^6, 19, 22^. In HBV, the *PreS1* domain of *S* ORF is found to be strictly constrained, whilst the *Spacer* domain of its overlapping *P* ORF is prone to non-synonymous mutations^19^. However, we argue that these outcomes of codon-based statistics might be misread, because the synonymous sites of overlapping regions, which are often non-synonymous in one of the reading frames, do not evolve neutrally. In fact, a decreased rate of synonymous mutations (*d*_S_) in overlapping versus non-overlapping regions has been demonstrated in various viral species^5, 6^. In such case, the codon-based estimation of selective pressure in overlapping genes is under question, because the neutral substitution rate *d*_S_ is occasionally underestimated. Thus, we propose the use of an accurate definition of neutral variants, as well as an improved classification of independent and joint amino acid mutations referring to previous work^18^. We applied the MK test to evaluate adaptive evolution of overlapping genes, because of its flexibility in dealing with any putative variants (e.g. INS, DNS, and even non-coding)^23^ as long as the neutral substitutions are well defined.

In conclusion, this study provides a framework to understand adaptive evolution of viral genomes, and improves current methodologies to better handle with genetic data in respect of gene overlaps. Using a classical statistical test of neutrality, this study reveals signatures of adaptive evolution in HBV proteins, thereby sheds a light into the exploration of virus-human co-evolution and future treatment to hepatitis B.

## Materials and Methods

### Data collection and pre-processing

The key word “Hepatitis B virus genome” was used to search against NCBI nucleotide database. The result was downloaded as fasta format, containing 8653 sequences. A filtering step was adopted to remove incorrect and low quality sequences, defined as: 1) sequence length <3100 or >3300 bp (out of range for a common and complete HBV genome); 2) sequences with >10 ambiguous bases; 3) redundant sequences. A total of 1888 sequences were removed in this step.

The circular genome sequences were modified to start with the ORF of *P* gene. All nucleotide sequence alignments in the present study were performed using ClustalW-MPI^52, 53^, with all parameters set as defaults. BioEdit^54^ was used to manually modify the mismatches of codons.

### Exclusion of inter-genotypic recombinants and population outliers

Inter-genotypic recombinants are potential admixture of genetically distant viral populations (in particular, each distinct HBV genotype is defined as one viral population), which largely bias the estimation of inner- and inter-genotypic diversities. To detect inter-genotypic recombinants, we adopted a fragment typing approach^25, 26^. First, the alignment of 6765 HBV sequences were devided into 250-bp fragments. Then, for each fragment, consensus sequences of 8 major human HBV genotypes (A–H) and 5 primate HBV species (chimpanzee, gorilla, gibbon, orangutan, woolly monkey) were generated based on subsets of the sequences with known genotypes (according to NCBI annotation). For the reason that majority of genotype B strains in public data were considered as B/C hybrid^26^, the consensus sequence of genotype B was calculated based on 32 pure B_j_ strains (AB010289–92, AB014366, AB073838, AB073842–58, AB106884–85, AB205121, D00329, D23677–79, D50521–22)^26^. The consensus fragmental sequences were used to construct blast database. Then, for each genome sequence, we performed BLASTN search to find the best hit for each 250 bp fragment and assign its putative “fragmental genotype” (Supplementary﻿ Table S1). Genomes with identical genotypes for all 250-bp fragments were considered as pure strains without inter-genotypic recombination, for example, “A1-A2-A3-A4-A5-A6-A7-A8-A9-A10-A11-A12-A13” for pure genotype A. To validate the method of detecting inter-genotypic recombinants and pure genotypes, we randomly select 24 pure strains (3 from each genotype) and 10 potential recombinants defined by fragment typing (Supplementary Table S2), and submit their sequences to jpHMM web-server, which is probabilistic model-based software to predict and visualize inter-genotypic recombinants^27^. Visualized results of four example strains were showed in Supplementary Figure S1. After excluding inter-genotypic recombinants and strains infecting non-human primates, 3947 sequences of human HBV genotype A-H were preserved (Supplementary Table S3).

Based on separate alignments of genome sequences per genotype, we further excluded 941 sequences with insertions or deletions relative to their own genotypic consensus sequence (Supplementary Table S4), to ensure the high quality of sequence alignment for further analysis. In the final step, we visualized the genetic distances within the 3006 sequences by PCA (performed using our in-house script in R language) and excluded 14 population outliers (Supplementary Figure S1, Supplementary Table S4). The outliers were determined by visual inspection and manual check on the values of principal components in each genotype.

### Population genetic analysis

Phylogenetic analysis of the 2992 HBV genomes were performed using MEGA5^55^. The genetic distance between sequences, measured by the number of substitutions per site, was constructed using the Maximum Composite Likelihood model. The phylogenetic tree was constructed using Neighbor Joining method^56^, with 100-time bootstrapping test.

Statistic *π* (pair-wise nucleotide differences) were calculated using DnaSP^57^ with a 100-bp sliding window at 50-bp step to measure the extent of genetic polymorphism within different viral genotypes. A standardized Z_*π*_ is then calculated as

1 $${{\rm{Z}}}_{\pi }=\frac{|\pi -\mu |}{\sigma },$$

where *μ* stands for the mean and *σ* stands for the standard deviation of *π* from all 100-kb windows. Derived alleles of a polymorphic site are defined as the alternative nucleotides distinguished from the dominant one. For example, at a single nucleotide polymorphic (SNP) site, 7 of 10 genotype H strains exhibits nucleotide residue A, another two has T and one has C. Therefore, A is the major allele of the SNP site, while T and C are two derived alleles, and their derived allele frequency (DAF) are 20% and 10%.

### Categorization of variants in overlapped regions

The variants were summarized into several categories according to their protein sequence outcomes: 1) non-overlapping non-synonymous (NNS) mutations; 2) independent non-synonymous (INS) mutations, which occur in overlapping ORFs, but only cause single protein alteration (for example, a non-synonymous mutation on *PreS1* but synonymous on *P*); 3) dual non-synonymous (DNS) mutations, which cause dual protein alterations from two different ORFs at the same time; and 4) synonymous mutations, which caused no amino acid change in either overlapping or non-overlapping regions.

The inner-genotype polymorphic mutations and inter-genotype fixed mutations were characterized using our in-house perl script. Mutations were categorized into four variant types described above by comparing the mutated codons with the consensus codons of its genotype, which represented the ancestral state of the polymorphic site (Supplementary Figure S3). Fixed mutations were determined similarly based on an alignment of eight HBV genotypic consensus genomes (Supplementary Figure S4).

### Analysis of positive selection during population differentiation

The present study used the MK test and its related statistics to measure the positive selection during genotypic differentiation. The original MK approach used the statistic *α* to measure the proportion of divergence driven by positive selection^24^. *α* is calculated as

2 $${\alpha }_{{\rm{X}}}=1-\frac{{D}_{{\rm{S}}}\cdot {P}_{{\rm{X}}}}{{D}_{{\rm{X}}}\cdot {P}_{{\rm{S}}}},$$

where *D* and *P* denotes the number of fixed and polymorphic mutations respectively, S denotes synonymous mutations and X denotes mutations putatively under selection (for example, X = INS, DNS or NNS). Here, to better visualize the positive selection on different categories of variants, we transformed *α* into the log-scale statistic *AS* (adaptive selection)

3 $$A{S}_{{\rm{X}}}={\mathrm{log}}_{2}(\frac{{D}_{{\rm{X}}}\cdot {P}_{{\rm{S}}}}{{D}_{{\rm{S}}}\cdot {P}_{{\rm{X}}}}).$$

If the evolution of X is not driven by selection, the ratio of fixed mutations and polymorphic mutations on X should equal that of neutral sites, therefore *AS*_X_ = 0. When *AS*_X_ > 0, positive selection drives the fixation of X and increase the proportion of fixed mutation between genotypes. *AS*_X_ < 0 suggests an opposite trend of adaptive evolution, where less fixed mutations are observed than the expected number. Confidence intervals of *AS*_X_ were computed from a non-parametric bootstrapping procedure as described previously^23^ to evaluate robustness of statistic *AS*_X_ based randomly selected genomic sites. *χ* ^2^ tests^30^ were performed on *D*_X_, *D*_S_, *P*_X_, *P*_S_ to infer the probability for the observed number of fixed mutations in X under neutral evolution.

## Electronic supplementary material

Supplementary Figures

Supplementary Tables
