# Supplementary material for: Adaptive evolution of proteins in hepatitis B virus during divergence of genotypes
Source: Sci Rep. 2017 May 16;7:1990. doi: 10.1038/s41598-017-02012-8 (PMC5434055; doi:10.1038/s41598-017-02012-8)
Supplement: Supplementary file 1 — Supplementary Figures [file 41598_2017_2012_MOESM1_ESM.pdf]

Supplementary figures

Adaptive evolution of proteins in hepatitis B virus during divergence of genotypes

Shengdi Li[1,2], Zhen Wang[1], Yixue Li[#,1,3], Guohui Ding[#,1,3]

[1]Key Laboratory of Computational Biology, CAS-MPG Partner Institute for Computational Biology, Shanghai Institutes for Biological Sciences, Chinese Academy of Sciences, 320 Yue Yang Road, 200031 Shanghai, China;

[2]University of Chinese Academy of Sciences, Beijing, China;

[3]Shanghai Center for Bioinformation Technology, Shanghai Industrial Technology Institute, 1278 Keyuan Road, 201203 Shanghai, China;

[#]Correspondence: Guohui Ding (gwding@sibs.ac.cn), Yixue Li (yxli@sibs.ac.cn).

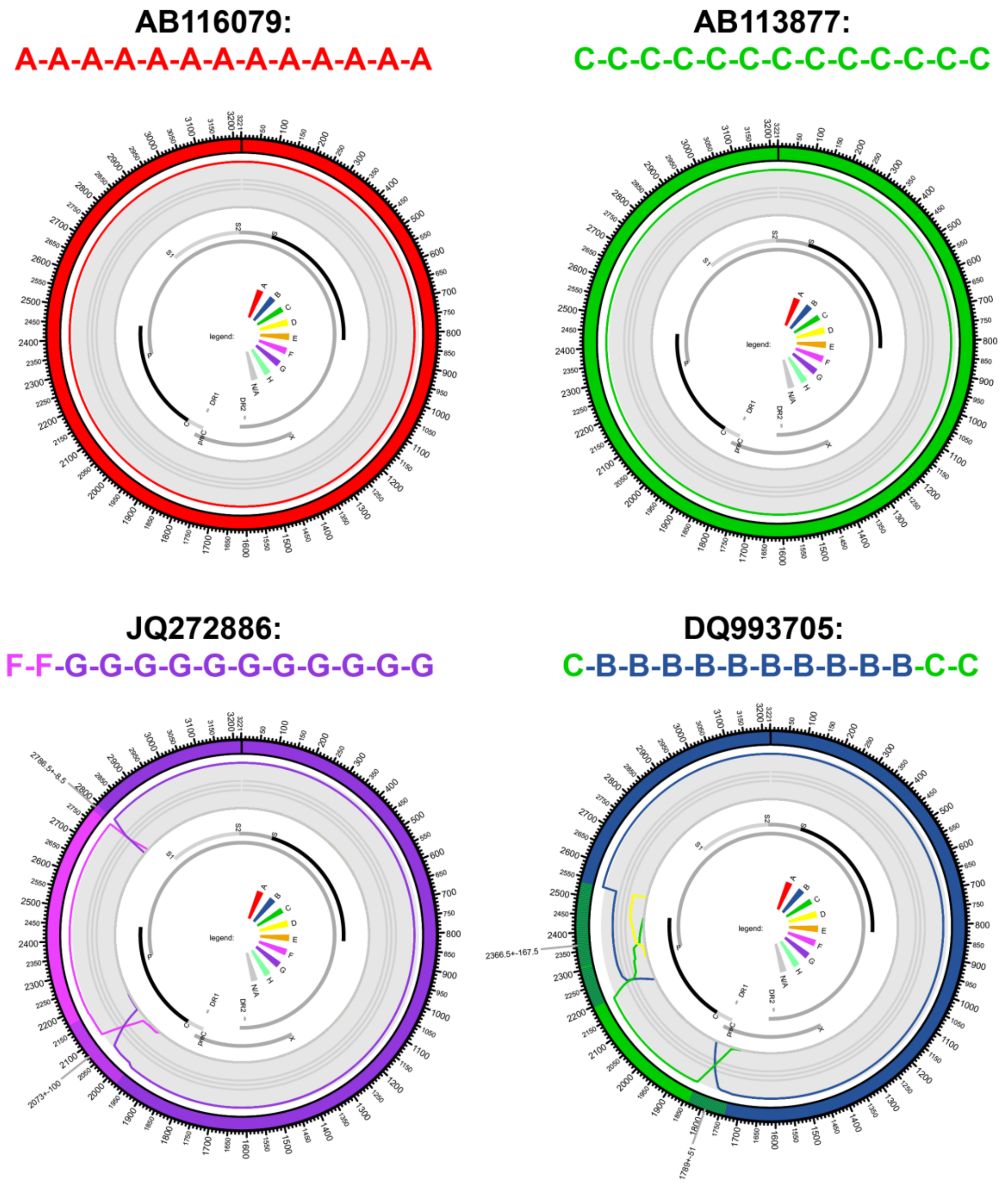

Supplementary figure S1. Visualization of pure-genotypic strains and inter-genotypic recombinants by jpHMM.

The genotype plots of two pure strains (AB116079, AB113877) and two inter-genotypic recombinants (JQ272886, DQ993705) generated by jpHMM. The fragmental genotypes (13 characters) predicted by fragment typing are displayed accordingly.

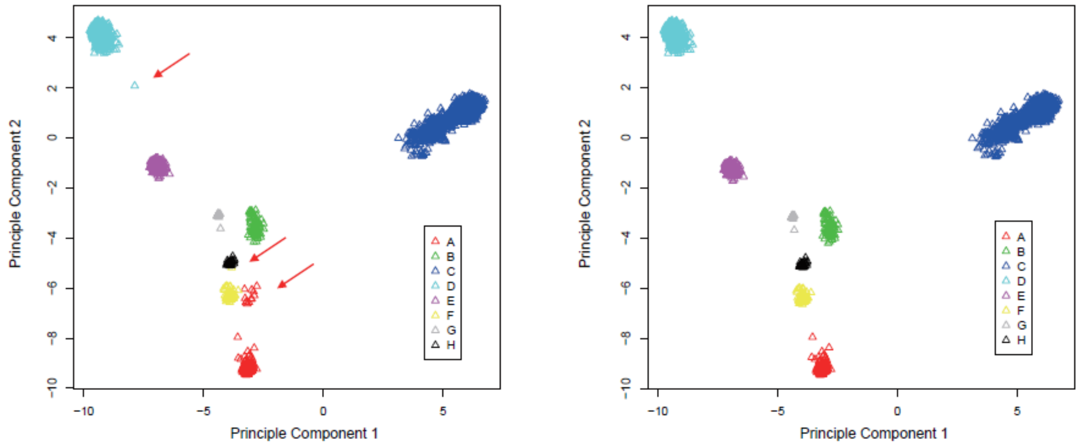

Supplementary figure S2. Removal of population outliers.

The genetic relationship of 3006 genomes before removing outliers (left) and 2992 genomes in final dataset (right) revealed by PCA. Population outliers were indicated by arrows.

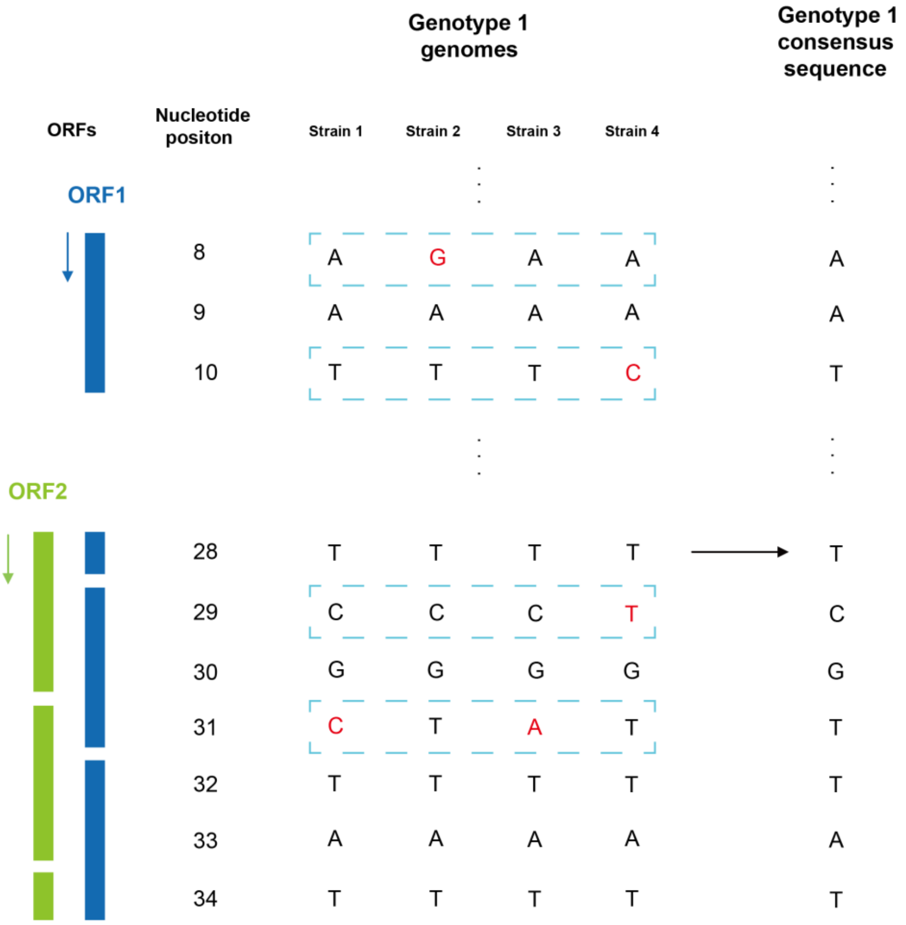

Polymorphic variant No.	Position	Variant	Codon change	Amino acid change	Variant type
No. 1	8	A->G	ORF1: AAT->GAT	ORF1: Asn->Asp	NNS
No. 2	10	T->C	ORF1: AAT->AAC	ORF1: Asn->Asn	synonsymous
No. 3	29	C->T	ORF1: CGT->TGT,<br>ORF2: TCG->TTG	ORF1: Arg->Cys,<br>ORF2: Ser->Leu	DNS
No. 4	31	T->C	ORF1: CGT->CGC,<br>ORF2: TTA->CTA	ORF1: Arg->Arg,<br>ORF2: Leu->Leu	synonsymous
No. 5	31	T->A	ORF1: CGT->CGA,<br>ORF2: TTA->ATA	ORF1: Arg->Arg,<br>ORF2: Leu->Ile	INS

Supplementary figure S3. Example of determining four categories of polymorphic variants.

This is an example on determination of NNS, DNS, INS and synonymous variants. The template dataset contains 4 strains from fictional genotype 1. Polymorphic sites are indicated in dashed boxes and variants distinguished from consensus sequence are marked as red. The codons of two open reading frames (ORF1 and ORF2) are showed as blue and green bars.

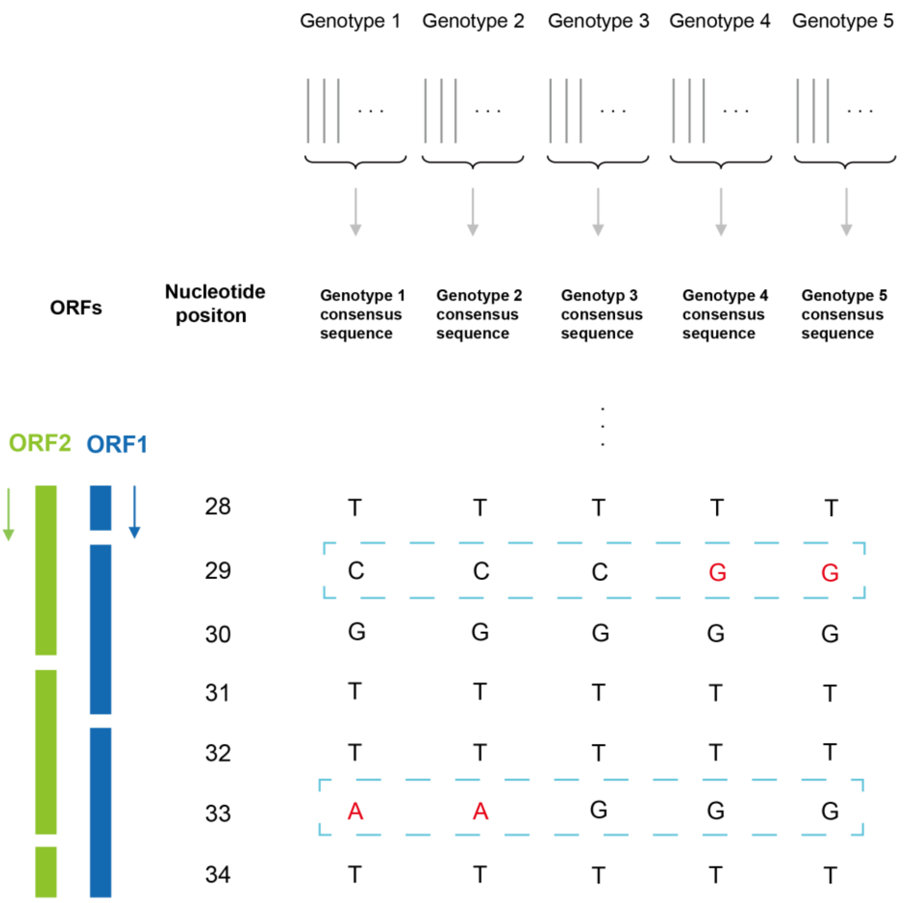

Fixed variant No.	Position	Variant	Codon change	Amino acid change	Variant type
No. 1	29	C->G	ORF1: CGT->GGT, ORF2: TCG->TGG	ORF1: Arg->Gly, ORF2: Ser->Trp	DNS
No. 2	33	G->A	ORF1: TGT->TAT, ORF2: TTG->TTA	ORF1: Cys->Tyr, ORF2: Leu->Leu	INS

Supplementary figure S4. Example of determining fixed variants.

The consensus sequences of each genotype were first generated and aligned. Fixed variants were then identified similarly as **Supplementary figure S3**.
